# A History of the High Operation for the Stone, by Incision above the Pubis; with Observations on the Advantages Attending on It; and an Account of the Various Methods of Lithotomy, from the Earliest Period to the Present Time

**Published:** 1820-01-01

**Authors:** 


*A History of the high Operation for the Stone, by Incision above the Pubis; with Observations on the Advantages attending on it; and an Account of the various Methods of Lithotomy, from the earliest Period to the present Time.* By J. C. CARPUE, F.R.S. &c. One Vol. Octavo, pp. 204, with plates. London, 1819.

WE do not conceive it necessary that a surgeon, who wished to introduce an improved bullet-forceps to the notice of his brethren, should write a professed history of Military Surgery on *that* account; nor can we think that Mr. Carpue was called upon to compile an "account of the *various* methods of lithotomy, from the earliest periods," in order to bring to our notice an operation which he saw Dr. Souberbielle perform in France, and Sir Everard Home in England, for the extraction of calculi from the bladder, by an incision above the pubis. Mr. Carpue, however, has thought otherwise; and as this is a mere question of policy, we shall not discuss it. It is not our intention, at present, to touch on any part of the *historical* matter in the volume before us, since it would be somewhat misplaced in a journal dedicated to the *progress*, rather than the *retrospect* of medical science.

When Mr. Carpue was in Paris, about two years ago, he was invited to see Dr. Souberbielle perform the operation in question, at the HÔTEL DES INVALIDES, on a gentleman, M. De Walfille, aged [illegible]4.

"Being placed as is usual in the operation for the stone, Dr. Souberbielle, having introduced a staff, made an incision in the perinæum, and into the membranous part of the urethra; he then introduced a director into the groove of the staff, which he withdrew. He now passed along the director, an instrument that had somewhat the form of a catheter, which was held by an assistant. The director was removed, and the instrument was suffered to remain in the bladder. The ligatures were taken from the feet of the patient, who was placed in a very different position. The operator made an incision in the integuments and fat, three or four inches in length, above the pubis. Dr. Souberbielle proceeded with great coolness, and extracted a large stone, then another, another, and another; he now drew forth a large quantity of small calcarious particles; this rendered the operation tedious, which the patient bore with great courage." 2.

This patient had a very good recovery. Dr. S. shewed Mr. Carpue several other patients on whom he had operated with success, in this way, and also performed it on the dead body before him. Mr. Carpue next practised on the dead subject himself, until he made himself perfect in the modus operandi. The operation performed by Sir Everard, from Mr. Carpue's suggestion, is very slightly glanced at in the work before us, and is, in fact, much more circumstantially detailed in our Third Number, page 451. The following is Mr. Carpue's recapitulation of the steps of this operation.

" A staff is introduced into the bladder first, an incision is made through the integuments of the perinæum, and a small incision into the membranous part of the urethra; a director is introduced into the bladder, upon the staff: the staff is withdrawn; the *sonde-de-dard* is introduced upon the director into the bladder: the director is now withdrawn; the *sonde-de-dard* is held by an assistant. An incision is then made, three or four inches in length, through the integuments of the abdomen. The trocar-bistouri is passed through the linea alba, close to the posterior part of the pubis. The concealed blade is opened, by means of which the lower part of the linea alba is divided. A probe-pointed bistouri is introduced through the opening which had been made by the concealed bistouri, into the lower part of the linea alba, and the incision is continued by means of this instrument. The operator takes the *sonde-de-dard* from the assistant with his right hand, and pushes it forward, by which means he elevates the bladder above the pubis. The assistant now holds the *sonde-de-dard*, and the surgeon, with his right hand, pushes the stilet, (which is contained in the cannula of the *sonde-de-dard)* through the superior and anterior part of the bladder; he takes hold of the end of the stilet with his left hand, and passes a probe-pointed bistouri along the groove, (which is in the anterior part of the stilet) and makes an incision in the superior anterior part of the bladder. He passes the index finger of the left hand into the bladder, by means of which he supports it. The stilet is withdrawn from the cannula of the *sonde-de-dard*, which is now lowered, and held by an assistant. The stone is now to be withdrawn with the finger and thumb, which if small, is done with great ease. If the bladder is large, a finger is introduced per rectum, by which the bladder is elevated, and the stone more readily found. If the stone should be in an excavation, and the bladder is not of a very large size, it may be discovered with the finger, by means of which the surgeon will know whether a scoop, or what kind of forceps is indicated." 169-171.

If the stone be large, forceps must be used; and if encysted, the cyst must be broken down. Souberbielle, when the stone is extracted, introduces a silver wire through the cannula of the *sonde-de-dard,* and passes it through the wound made in the linea alba; this is held while the *sonde-de-dard* is withdrawn. A flexible gum catheter is now passed into the bladder, through the wound in the membranous part of the urethra, by means of this wire, which last is then withdrawn. The catheter is confined in this situation by tapes passed round the thighs and pelvis, and a bladder is tied to it to receive the urine. In respect to dressings, the following directions are given by our author.

"A piece of soft linen, half an inch wide, and six or eight inches long, is to be introduced, by means of a pair of forceps, into the bottom of the bladder; the edges of the wound are to be covered with lint, to prevent the urine excoriating the parts; the linen is to be allowed to pass over the pubis on either side, and by this means the portion of urine which is not carried away by the catheter, will be carried off by means of the linen."* 172.

Great care is to be taken that the catheter is kept open; and on that account a stilet should occasionally be passed. On the third day the linen may be taken from the bladder, as by that time the greater part of the urine will pass by the catheter, and the wound will be suppurating.

Mr. Carpue offers the following reasons for giving preference, *in most cases,* to the high operation.

"1. Because it is generally performed in less time than the lateral operation.

"2. There is less pain.

"3. There is no fear of a fatal hæmorrhage.

"4. There is no division of the prostate, nor of the inferior part of the bladder, nor is there any danger of wounding the rectum.

"5. The stone, if of a certain size, cannot be extracted by the lateral, but may be extracted by this method.

"6. A small stone is more readily discovered by this method than by the lateral.

"7. If the stone breaks, the particles can be extracted with more certainty than in the lateral operation.

"8. If the stone is concealed in a cyst, the cyst can be destroyed and the stone extracted, as is proved in Sir Everard Home's case. There is also no danger of including part of the bladder with the stone, in endeavouring to extract it, nor any danger of a fistulous opening after the operation.

"9. In case there should be any disease of the bladder, it can be examined, and proper means prescribed for the cure.

"10. In patients where the staff cannot be passed, in consequence of stricture or disease of the prostate, or where the calculus is of a certain magnitude, there is no choice of the mode

---

* "If you place a piece of linen in the bottom of a cup filled with water, and let the ends rest upon a plate, in three or four hours, the whole of the water will be in the plate."

of operation. *Either the high operation must be performed,* or the patient is doomed to linger out a life of wretchedness." 173, 4, 5.

Mr. Carpue considers the operation inapplicable in corpulent subjects, or where there is such organic disease of the bladder as may prevent the *sonde-de-dard* from raising its fundus above the pubis.

Thus far we have proceeded in a strictly *analytical* course; but, however reluctantly, we must occasionally assume the *censorial* character, otherwise an important object of the Journal (though *comparatively* a minor one) would fall to the ground. We shall not, however, lose sight of the rocks on which so many of our predecessors foundered. The flattery of the deceitful Syren, and the cannibalism of the unrelenting Cyclops, are equally dangerous, and equally to be avoided. He who for self-interest would pour the incense of adulation at the shrine of rank or power, is as much a dastard as the man who, from malice or revenge, would use the dagger, because he is himself secured by the mask!

In the first place, we object to the air of *novelty* with which this operation is brought forward, both because it differs in no essential point from that of Frere Cosme, and because we understand, from good authority, that the identical operation of Souberbielle has been performed, on an average of from five to seven times annually in Paris for many years past, *in cases where the lateral operation was deemed objectionable or inefficient, in consequence of the size of the stone or other circumstance.* We were struck also with an inconsistency in the work under review. At page 129, Mr. Carpue gives an extract from Frere Cosme, descriptive of the high operation performed by Souberbielle; yet, in the *Preface,* he states, when called upon to sign the certificate, that he "did not understand the operation." Did this proceed from the preface having been printed off, before he discovered that Frere Cosme had anticipated his friend Souberbielle? At page 168, it is inferred, that Sir Everard Home too was ignorant that, in the modern high operation, a cut was made in the perinæum; an inference which we can hardly admit, when every student who reads page 696 of Cooper's Surgical Dictionary, [3d Edit.] will see Frere Cosme's operation, agreeing, in all essential respects, with that of Souberbielle.

It may seem somewhat hardy in *us* to take a professed teacher of anatomy to task on *that* branch of medical science; yet, certain passages in the work before us, have recalled to our minds the favourite researches of our youth, the fruits of which, we hope, are not quite withered and decayed by the hand of Time.

"If, for a moment, (says Mr. Carpue) we consider the situation of the bladder, and the parts *connected with it*, such as the levatores ani, the obturatores interni, the coccygean muscles, the muscles from the rami of the ischium, we shall find that these muscles act on a part," (*i. e.* the *bladder*) "which is but slightly attached to the pubes by cellular substance, and by the lateral ligaments; these I find, by repeated dissections, are seldom the same in different subjects. I say, if we consider the action of these muscles, the situation of the bladder must vary under different circumstances." 134.

Whether, during the revolutions of the last eventful twenty years, the anatomy of the pelvis may have changed, with crowns, kingdoms, and territories, we cannot pretend to say; but, if our memories do not fail us, our early dissections taught us that the *obturatores interni*, instead of being attached to the bladder, were separated from it by the levator ani and a strong fascia, and that they passed *out of the pelvis* to be inserted into the pit of the trochanter. The same dissections had led us to consider the *coccygean muscles*, as separated from the bladder by the rectum and its contents; and that, instead of being attached to the urinary receptacle, they were inserted into the sacrum and os coccygis. We used to trace the insertions of the levatores ani into the sphincter ani, the membranous part of the urethra, and the prostate gland; but, as for the other "muscles, from the rami of the ischium," stated by Mr. C. to be connected with the bladder, they were, in those days, no where to be found.

Mr. Carpue seems to dread a fatal hæmorrhage from some branch of the pudic artery distributed on the prostate gland, in the lateral operation. As it happens, that in about one case in fifty, the pudic artery itself takes a course along the prostate gland to get through the labyrinth into the body of the penis, it is probable that Mr. Carpue has mistaken some of the large veins for arteries, since a glue injection passes readily into the veins from the arteries.

We should not, probably, have indulged in these few anatomical criticisms, had not the work proceeded from a teacher of that science; and consequently, where any errors appear in a more glaring light, than in the publication of a plain practical surgeon, who might be supposed to have forgotten the minuter features of his original acquirements in the dissecting room.*

We shall now adduce our objections to the high operation, in the order of Mr. Carpue's list of advantages, quoted a page or two back, and which the reader will please now to turn to, that we may not have to recapitulate them here.

1. The lateral operation does *not* require a longer time than the high process; nor, in general, near so long a time. Five or ten minutes are usually sufficient for the operation, as now performed; and we conceive, that it would require this space to put M. Souberbielle's instruments in a proper state of arrangement. Sir Everard Home's operation required, by Mr. Carpue's account, seventeen minutes. We have heard that the time was longer than here stated.

2. It is very questionable, if the pain be less in the high, than in the lateral operation.

3. In respect to the hæmorrhage, we may observe that, to a certain extent, it is advantageous, in the lateral operation, to divide those branches of the pudic artery which ramify on the prostate gland. If the operation be performed in a proper manner, the chances of bleeding, from an irregularity in the distribution of the pudic artery, are not greater than in the high operation, from the pudic coming off from the obturator; and, in that case, running on the upper part of the bladder. Indeed, it would appear that Mr. Carpue is not aware of the true cause of fatal hæmorrhage in the lateral operation. It arises from the pudica, on the ramus ischii, being cut by a push of the gorget, or by the bistoire cachée being opened so wide as to rasp along the bone.

4. Mr. Carpue's fourth advantage, in the high operation, is of the *negative* kind; namely, that there is no division of the prostate gland, nor danger of wounding the rectum. To this we may reply, that, since the days of Cheselden, the success of the lateral operation has been considered as mainly owing to the very circumstance of the wound being in the prostate gland and sphincter of the

* The Editor has reason to believe, that Mr. Carpue entertains some peculiar opinions respecting the effect which muscles, not strictly speaking connected with the bladder, have on the relative position of that organ. He thinks, for instance, that muscles going from the pelvis to the thigh, will influence, while acting, the site of the bladder. But Mr. Carpue should have been more explicit on these points. Into how many errors, controversies, and quarrels, have men been led, by want of accuracy and perspicuity in language!

bladder. In respect to the wounding of the rectum, we may observe that, were the accident to take place, the consequences would not be nearly so dangerous as a wound of the colon or rectum, *adherent to the fundus of the bladder*, which they not unfrequently are, in the inflamed bladder.

5. The fifth advantage is true, but not new.

6. New; but doubtful in respect to its truth.

7. We are surprised that Mr. Carpue should suppose that the fragments of a broken stone are more readily extracted in the high than in the lateral operation, when the very *reverse* was one of the principal reasons why the high operation was given up. What can be more easy than the washing out of the particles in the lateral operation?

8. We know that the stone was supposed to be encysted, in Sir Everard Home's case; but we strongly suspect that it was only a contraction of the bladder; for such is generally the case, in what are called encysted stones in children. We have seen a preparation called an encysted stone, from the body of a child; but, instead of a cyst, as is found in old men, it was the bladder itself closely investing the stone. It is very easy to explain this. By the cut in the perinæum, the urine, in most cases, immediately escapes, particularly in children, where the detrusor urinæ is in violent action during the operation, and, as soon as the water is evacuated, clings with spasmodic force to the stone, thus forming what is falsely termed a cyst.

As to the danger of a fistulous opening remaining after the lateral operation, the same objection is stronger against that above the pubes. Even in Sir E. Home's case, the patient was long of recovery, in consequence of the difficulty of closing the fistulous opening in the belly; whereas, we have reason to know, that the opening in the perinæum was closed in a very few days.

9. The ninth advantage we really are unable to perceive, unless an *illuminator* be invented, for the purpose of examining the bladder through the small opening above the pubes.

10. We have said before, that the operation has long been performed, several times annually, in Paris, and taught in the public schools there. It was performed lately by M. Dupuytren, and so it is by all the regular surgeons, when deemed eligible. The cases in which it is preferred, are those wherein the lateral operation would be dangerous, as where the stone is very large, or where there is much disease of the prostate gland. Such cases are so deplorable, that a desperate operation, to relieve the miseries of the patient, is admissible. We know that, at one time, M. Souberbielle himself, who is one of the old class of lithotomists, performed the high operation in preference to the lateral, *only* in such cases as above described. Indeed, Mr S. Cooper, when in Paris, saw M. Souberbielle perform the *lateral* operation on a gentleman seventy years of age; and a friend of ours saw him commence the lateral operation on a patient; but having gone through the first steps, he stopped short, saying, "Ah! la pierre est trop grande," and then proceeded to extract the stone from an opening above the pubes. It would appear, from the ages of M. Souberbielle's patients, as given by Mr. Carpue [from 50 to 86, all except one] that they had arrived at that period of life when the prostate gland is almost invariably diseased, when previous irritation had long existed in the bladder. Such cases are, of course, the legitimate subjects for the high operation; and in *such cases,* we object not to the operation, but only to the attempt to substitute it for the lateral in *all* cases.

The following objections to this operation, excepting in the cases alluded to, may serve as a set off against Mr. Carpue's list of advantages.

1. *The danger of wounding the Peritoneum.* This is more likely to happen in, as it is called, the *improved* operation of Frere Cosme, than in the old one; because the wound in the perineum permits the escape of the urine, the consequence of which is, the contraction of the bladder on the stone. If the latter be small, the bladder will come close on the pubes, and be in great danger of being wounded by the *sonde-de-dard.* This may be easily demonstrated on the dead body, where the bladder is much contracted. The danger of wounding some of the intestines, in such cases, will also be manifest.

2. There is a chance of a fistulous opening remaining in the belly.

3. But the greatest objection of all, is the danger of extravasation of urine between the muscles of the abdomen and the peritoneum.

4. All the first surgical authorities in the world are decidedly against the high operation *in common cases.* Even Frere Cosme, the inventor of the new operation, never performed it where he could do the *lateral* operation. When, indeed, we see an expert surgeon perform the *latter* operation, and observe that the patient is generally off the table in six or eight minutes, and walking about in a month, we should be cautious how we speculate on farther improvements. Let us bear in mind the inscription on the tomb.

"I was in *good* health—took physic to make it *better*—and here I am!"

